# Announcing sustainable microbiology: How microbes make a sustainable world

**DOI:** 10.1093/sumbio/qvae001

**Published:** 2024-01-24

**Authors:** David A Pearce

**Affiliations:** Department of Applied Sciences, Faculty of Health and Life Sciences, Northumbria University at Newcastle, Northumberland Road, Newcastle-upon-Tyne, NE1 8ST, United Kingdom; E-fellow, British Antarctic Survey, Natural Environment Research Counicil, High Cross, Madingley Road, Cambridge, CB3 OET, United Kingdom

Microorganisms are at the very heart of sustainability. In their very basic form, microorganisms could be considered as small bags of enzymes able to respond to environmental signals (with viruses able to influence such interactions). However, they form an integral part of the Earth System through biogeochemical cycling; they degrade and recycle the results of human activity; they are essential to achieve global food security and their activity is known to influence global climate processes. Critically, their role and activity may alter in a changing environment, including in ways that are as yet undefined. Hence, the potential for microbiology to solve some of the world's greatest challenges is vast.

Applied Microbiology International (AMI) believes that global interdisciplinary teams are key to solve many global challenges. As such, AMI has been designed with a clear purpose: to bring the microbiology community closer together, enabling meaningful global interdisciplinary collaboration to advance the scientific impact, with microbiology at its core. Microbiology as a subject is very broad, differentiated into disciplines such as molecular biology, genetics, multi-omics, and bioinformatics, all of which have grown from the discipline and established themselves as subjects in their own right. However, the subject of microbiology continues to evolve, driven through innovation and technological advances both within and outside the discipline.

All encompassing, microbiology holds enormous potential to alter the course of many human challenges as exemplified by the UN Sustainable Development Goals (SDGs). Hence, AMI is now focused on eight of the UN SDGs where microbiology can play a leading or central role (i.e. no poverty, zero hunger, good health and wellbeing, clean water and sanitation, affordable and clean energy, climate action, life below water, and life on land). As a journal, we will focus on novel research and literature reviews on the role of microbes in achieving a sustainable world.

It is a great pleasure to write this editorial, published alongside our first content, to introduce some context for the launch and share our vision for the future. We are delighted to be launching in partnership with Oxford University Press and welcoming a new journal to the AMI family.

Our journal will be open access, ensuring maximum reach and visibility of authors’ work. We will take a novel approach, drawing together publications from different sectors, including policy and industry, as well as academia, with the aim of stimulating interdisciplinary collaboration that leads to scientific advancement. Policy content will highlight how new microbiological understanding has the potential to support and improve policy and decision-making; or deliver specific impact on sustainability. Industry Spotlight articles will showcase new products or services specifically applying microbiology to the UN SDGs, focusing on the underlying science. We will also publish original research and high-quality *reviews*. Our scope is therefore broad, covering all microbes in their full diversity and complexity, in which the authors can describe the ability of the scientific observations to help deliver specific impacts on sustainability. Published articles will contain a ‘sustainability statement’ making such links clear to readers, be they academics, working in industry, practitioners, or at the policy level and helping authors to demonstrate the potential impact of their work.

We have assembled an international editorial board of subject experts, who are committed to providing rapid and constructive peer review of submitted manuscripts. We will continue to recruit additional editors as the journal grows and to ensure the broadest subject coverage, always with consideration of gender, geographical, and career-stage diversity. Please do feel free to reach out to editors who are aligned with your area of study to discuss any questions you may have or to provide feedback that will help us to shape the journal and conversation around microbiology for sustainability.

As a society-owned journal, published by a not-for-profit publisher, all profits from the journal go to supporting the community and furthering science. Surplus from the journal will pay for a variety of grants available to the AMI community. To give something back to our authors and ensure they can join our AMI community and benefit from all the support they enable us to offer our members, we are offering all published authors a year's free membership.

Much has already been said about the need for increased representation of microbiology at the policy level, including most recently its disappointing omission from COP discussions [Gewin, V. Microbiology must be represented at climate change talks. Nat Microbiol 8, 2238–2241 (2023). https://doi.org/10.1038/s41564-023-01534-4]. Our vision is that Sustainable Microbiology will help to stimulate research, open-up the conversation, draw attention to policy-level work being carried out in this field, foster collaborations between academia and industry and clearly demonstrate the crucial role microbes have to play in improving global economic, social, and environmental sustainability. We have spent time over the past year meeting our community of future authors, reviewers and readers at conferences and symposia. We have been delighted by the positive reaction to the journal's launch. Whilst there still may be much work to do in achieving the UN SDGs, this is a community that is working hard on these challenges. We are ready to support you and showcase your important contributions.

Microbiology influences every aspect of our daily lives, so we welcome contributions from all areas of microbiology at all scales: from the planetary to the molecular, from agriculture, aquaculture, and food production to veterinary science, medicine, epidemiology, and disease control and from pure to applied sciences. We welcome manuscripts with a strong microbiology component, addressing at least one of the AMI target UN SGDs, which is expected to have a significant impact on our understanding and/or application of microbiology to address or solve these goals.

